# Photofermentative production of poly-β-hydroxybutyrate (PHB) by purple non-sulfur bacteria using olive oil by-products

**DOI:** 10.1186/s40643-025-00856-x

**Published:** 2025-03-24

**Authors:** Gianmarco Mugnai, Luca Bernabò, Giulia Daly, Elisa Corneli, Alessandra Adessi

**Affiliations:** 1 Department of Agriculture, Food, Environment and Forestry (DAGRI), University of Florence, Piazzale Delle Cascine, 18, 50144 Florence, Italy; 2 Department of Agricultural, Food and Environmental Sciences, University of Perugia, Borgo XX Giugno, 74, 06121 Perugia, Italy; 3 PhotoB. Srl, Via Montecalvi, 3, San Casciano in Val Di Pesa, 50026 Florence, Italy

**Keywords:** *Rhodopseudomonas palustris*, *Cereibacter sphaeroides*, *Cereibacter johrii*, Ingested pâté olive cake (IPOC), Biopolymers, Resource recovery

## Abstract

This study evaluated the ability of six purple non-sulfur bacteria (PNSB) to convert olive oil by-products into poly-β-hydroxybutyrate (PHB). Strains were first independently cultivated in synthetic media with different carbon sources (acetic, lactic and malic acid) to assess their physiology and PHB production. Subsequently, their growth and PHB production using ingested pâté olive cake (IPOC) as a substrate were investigated. Transmission electron microscopy (TEM) observations were conducted on strains cultivated on IPOC to investigate their cell morphologies and inclusion bodies presence and size. *Rhodopseudomonas palustris* strains accumulated up to 6.8% w PHB/w cells with acetate and 0.86% w PHB/w cells with a daily productivity of 0.54 mg PHB L⁻^1^ culture d⁻^1^ on IPOC. In contrast, *Cereibacter johrii* and *Cereibacter sphaeroides* reached 58.64% w PHB/w cells and 65.45% w PHB/w cells with acetate, respectively, while *C. sphaeroides* achieved 21.48% w PHB/w cells and a daily productivity of 10.85 mg PHB L⁻^1^ culture d⁻^1^ when cultivated on IPOC. All strains exhibited growth and PHB accumulation in both synthetic media and IPOC substrate. Specifically, *R. palustris* strains 42OL, AV33 and CGA009 displayed growth capability in all substrates, while *C. johrii* strains 9Cis and PISA 7, and *C. sphaeroides* F17 showed promising PHB synthesis capabilities. TEM observations revealed that *R. palustris* strains, with smaller cell and inclusion body sizes, exhibited lower PHB accumulations, while *C. johrii* and *C. sphaeroides* strains, characterized by larger cells and inclusion bodies, demonstrated higher PHB production, recognizing them as promising candidates for PHB production using olive oil by-products. Further investigations under laboratory-scale conditions will be necessary to optimize operating parameters and develop integrated strategies for simultaneous PHB synthesis and the co-production of value-added products, thereby enhancing the economic feasibility of the process within a biorefinery framework.

**Graphical Abstract:**

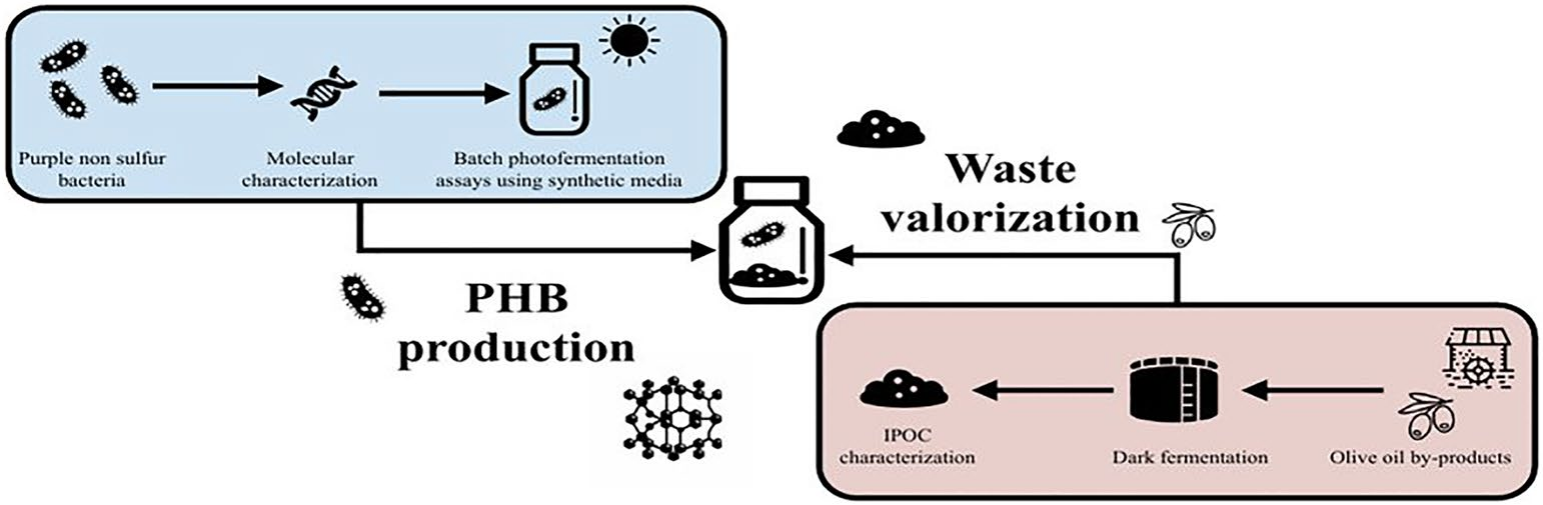

**Supplementary Information:**

The online version contains supplementary material available at 10.1186/s40643-025-00856-x.

## Highlights

- Growth and poly-β-hydroxybutyrate (PHB) production of Purple Non-Sulfur Bacteria (PNSB) on synthetic media predict their performance on olive oil by-products
- Ingested pâté olive cake (IPOC) is a suitable substrate for PHB production
- *R. palustris* strains utilized IPOC for cell growth, whereas *C. johrii* and *C. sphaeroides* synthesized PHB
- 21.48% PHB/cell weight was accumulated by *C. sphaeroides* F17 on IPOC
- Growth data and PHB production of the poorly described specie *C. johrii* are reported

**Supplementary Information:**

The online version contains supplementary material available at 10.1186/s40643-025-00856-x.

## Introduction

Third-generation feedstock, such as microbes, are emerging as promising biopolymer sources to replace petrochemical plastics, offering significant advantages by not competing with human food or animal feed and not requiring arable land or freshwaters resources (Amadu et al. 2021). A sustainable and eco-friendly type of microbial bioplastics is polyhydroxyalkanoates (PHAs), which are biodegradable polymers obtained from microbial fermentation. Poly-β-hydroxybutyrate (PHB) is the most extensively studied PHA and can be produced by a wide variety of bacterial strains, using renewable biomasses as substrates and is degradable by several aerobic and anaerobic microorganisms (McAdam et al. 2020; Monroy and Buitrón 2020). PHB exhibits mechanical properties similar to conventional thermoplastics (Monroy and Buitrón 2020; Sirohi et al. 2021), with a melting temperature of 177 °C, a glass transition temperature of 2 °C, a tensile strength of 43 MPa and approximately 60% crystallinity (McAdam et al. 2020; Sirohi et al. 2021). These physical and chemical properties demonstrated the wide range of possible applications: in agriculture (as fertilizer or for agrochemicals release), in medical sector (such as in surgical sutures and long-term drugs carriers) and in packaging industries (such as molded containers and food packaging materials) (McAdam et al. 2020; Monroy and Buitrón 2020; Sirohi et al. 2020, 2021; Montiel-Corona and Buitrón 2021).

A group of photosynthetic bacteria, called Purple Non-Sulfur Bacteria (PNSB), are capable of accumulating PHB (Adessi et al. 2017; Monroy and Buitrón 2020). The most studied PNSB species for PHB production include *Cereibacter sphaeroides (formerly Rhodobacter sphaeroides) Rhodopseudomonas palustris*, *Rhodobacter capsulatus*, *Rhodobacter sulfidophilus* and *Rhodospirillum rubrum* (Argun and Kargi 2011; Adessi et al. 2017; Hördt et al. 2020). The new genus *Cereibacter* has been reported according to Hördt et al. (2020); therefore, in this work, all genera and species will be referred to the current nomenclature, even when reporting the literature published before 2020.

Microbial PHB is produced as storage compound during starvation and accumulated in intracellular inclusions (McAdam et al. 2020; Monroy and Buitrón 2020; Fradinho et al. 2021). Several factors affect PHB production, including the type of strain, substrate, temperature, C/N ratio, nutrient concentrations, light fluxes and pH (Sedlacek et al. 2019).

During the photofermentation process, PNSB can produce hydrogen or PHB using carbon substrates, including organic acids, carbohydrates or wastewaters and effluents deriving from other processes, such as dark fermentation (Argun and Kargi 2011; Adessi et al. 2017; Monroy and Buitrón 2020). The sustainability of fermentative process mainly depends on the type of substrate employed as the carbon source (Adessi et al. 2017; Sirohi et al. 2020; Bhatia et al. 2021; Montiel-Corona and Buitrón 2021). Agricultural and food-processing residues represent low-cost, abundant, and renewable biomasses, providing a useful substrate for microbial growth due to their non-toxic properties and high nutrients and carbon content, including volatile fatty acids (VFAs), simple sugars, and alcohols (Adessi et al. 2017; Sirohi et al. 2021). Since the carbon source for bacterial PHB production accounts for about half of the total production costs (Brown et al. 2020), using these feedstocks as substrate can significantly reduce economic costs and environmental impact, thereby enhancing the profitability and sustainability of PHB production (Adessi et al. 2017; Monroy and Buitrón 2020; Sirohi et al. 2020, 2021; Bhatia et al. 2021). The most investigated no-cost agro-industrial wastes for the PHB production using PNSB includes lignin (Brown et al. 2020), agro-industrial residues (Corneli et al. 2016), cheese whey agro-industrial byproduct (Carlozzi et al. 2021), winery wastewater (Policastro et al. 2020), municipal solid waste (Luongo et al. 2017), digested sludge (Touloupakis et al. 2023) and olive mill waste water (Carlozzi et al. 2019b). These studies demonstrate the metabolic flexibility of PNSB in efficiently using different waste streams, highlighting their role as a promising ecofriendly biotechnological tool for waste valorization within a bio-based and circular economy framework.

The effluent from the olive-oil industry is consider one of the most polluting wastes in the Mediterranean basin due to its high salt concentration (EC 5–10 mS cm^−1^), high acidity (pH 4–5), high biological and chemical oxygen demand (BOD and COD of 100,000 and 220,000 mg L^−1^, respectively), and high content of polyphenols (Aharonov-Nadborny et al. 2018). The main by-products generated from olive oil process are olive mill wastewater (OMW) and olive pomace or pâté olive cake (POC) (Foti et al. 2022). Although recent studies have demonstrated that OMW is an interesting substrate for green energy production (Mugnai et al. 2021) and biopolymers (Carlozzi et al. 2019b; Corneli et al. 2016; Padovani et al. 2016), the application of olive cake as substrate for photofermentative processes is still lacking. POC generated by olive oil extraction using a multiphase decanter, contains high level of bioactive compounds such as fatty acids, proteins, and carbohydrates, representing a matrix for biotechnological applications in a biorefinery perspective (Foti et al. 2022). These advantages led to the use of POC as a valuable feedstock for anaerobic digestion, which can be conducted in one- or two-phase system to produce biogas and digestate. In a two-stage process, the initial stage produces an ingestate, called ingested pâté olive cake (IPOC), enriched in organic acids, such as acetic, propionic, and butyric acids, which serve as essential carbon and energy sources to the metabolism of PNSB.

The aim of this study was to select PNSB strains able to produce PHB using olive oil by-products as feedstock for sustainable bioplastics production. To achieve this goal, the experimental plan was divided into two main steps. First, the molecular identification and physiological characterization of six PNSB strains were performed using a synthetic growth medium with different single carbon sources (acetic, lactic and malic acid), evaluating their biomass and PHB production. Second, the growth capability and PHB production of the six selected strains were investigated using IPOC as substrate.

The strains used in this study include *R. palustris* strain 42OL, strain AV33 and CGA009, selected based on prior knowledge of their metabolic versatility and ability to use wastes for growth (Bianchi et al. 2010; Adessi et al. 2012, 2018), while three additional strains (*C. sphaeroides* strain F17, *C. johrii* strain PISA 7 and strain 9Cis), never tested before, were selected considering the different ecological niches from which they were isolated, spanning from different wastewater treatment plants (F17 and 9Cis) to nature reserve lacustrine area (PISA7). The ability to thrive under different environmental conditions, including limited nutrient availability, confers a competitive advantage and drives the development of distinct metabolic traits influenced by their ecological niches. We hypothesize that the ecological niche of isolation and the life-history strategies of each strain represent critical factors that could contribute to the identification and selection of new promising strains for PHB accumulation from agro-industrial waste.

## Materials and methods

### Strains identification

Relevant information on PNSB strains used in this study is listed in Table 1. The selection criteria for the strains investigated in this study were based on prior knowledge of their metabolic versatility and the ecological niches of their isolation, which could confer different metabolic traits. All strains are preserved in agar slants in the collection of Department of Agriculture, Food, Environment and Forestry (University of Florence, Italy). After DNA extraction, bacterial 16S rRNA region (V3-V4), were amplified by GeneAmp PCR System 9700 (Applied Biosystem, UK) using the following primers: 27F and 1837R (Marchesi et al. 1998). The DNA was subsequently purified using the Genejet PCR purification kit (Thermo Fisher Scientific Inc.) and its purity assessed spectrophotometrically (NanoDrop spectrophotometer ND-1000, Thermo Scientific, Wilmington, USA). The rRNA sequences were obtained by an external sequencing service (CIBIACI, Florence, Italy) and identified against the NCBI nucleotide sequence database. The most similar sequences were exported, and the sequence alignment (Muscle) was generated using Mega11 (Tamura et al. 2021). Phylogenetic tree reconstruction was performed using the neighbor-joining method (Saitou and Nei 1987). The robustness of the phylogenetic inference was estimated using the bootstrap method with 1,000 pseudo-replicates.

**Table 1:** List of used strains, isolation substrates and localities

Species	Isolation substrates	Isolation locality
*Rhodopseudomonas palustris* strain 42OL	Sugar refinery waste treatment pond	Castiglion Fiorentino, Arezzo, Italy
*Rhodopseudomonas palustris* strain AV33	Trophic lake	Averno, Italy
*Rhodopseudomonas palustris* strain CGA009	Chloramphenicol-resistant derivative of R. *palustris strain* CGA001	American Type Culture Collection (ATCC), US
*Cereibacter johrii* strain 9Cis	Dairy wastewater	Bologna, Italy
*Cereibacter johrii* strain PISA 7	Lake	San Rossore, Pisa, Italy
*Cereibacter sphaeroides* strain F17	Wastewater lagoon	Florence, Italy

### Inoculum and batch photofermentation assays using synthetic media

Photofermentation was carried out using pure cultures of each strain. The strains were previously activated in 250 mL bottle at 25 °C with a light intensity of 150 µmol (photons) m^−2 ^s^−1^ using malic RPN medium: malic acid 2.0 g L^−1^; yeast extract 0.2 g L^−1^; K_2_HPO_4_ 0.5 g L^−1^; K_2_PO_4_ 0.3 g L^−1^; MgSO_4_٠7H_2_O 0.4 g L^−1^; NaCl 0.4 g L^−1^; CaCl_2_٠2H_2_O 0.075 g L^−1^; ferric citrate 0.005 g L^−1^; NH_4_Cl 0.5 g L^−1^. Trace elements were supplied by adding 10 mL per liter of a solution containing: ZnSO_4_٠7H_2_O 10 mg L^−1^; MnCl_2_٠4H_2_O 3 mg L^−1^; H_3_BO_3_ 30 mg L^−1^; CoCl_2_٠6H_2_O 30 mg L^−1^; CuCl_2_٠2H_2_O 1 mg L^−1^; NiCl_2_٠6H_2_O 2 mg L^−1^; NaMoO_4_٠2H_2_O 30 mg L^−1^. All the above cited salts were purchased from Sigma Aldrich (USA).

The pH of the medium was adjusted to 6.8 using 2M NaOH, both prior to and following autoclaving, with measurements performed using a pH-meter (XS instruments, Italy).

The strains were grown until reaching the cell concentration of 400 mg L^−1^ to carry out the subsequent inoculation in RPN media containing different carbon source: acetic acid (3.6 g L^−1^) and lactic acid (1.8 g L^−1^). The photofermentation batch assay was carried out in anaerobic conditions using rubber-stoppered glass bottles (100 mL). Each bottle was filled with 100 mL of PRN medium with an initial cell concentration of 100 mg L^−1^ as cell dry weight (CDW). Bottles were kept at 25 °C with a light intensity of 150 µmol (photons) m^−2^ s^−1^, supplied by a warm white LED lamp, for 14 days. Samples (2 mL per replicate) were collected 3 times per week, from the beginning to the end, for determining biomass growth by spectrophotometric measurements of the optical density at 660 nm (OD_660_) and bacteriochlorophyll *a* (BChl *a)* content. At the end of the experiments, samples were collected to determine CDW (15 mL per replicate) and aliquots (60 mL per replicate) stored at – 20 °C until the determination of the intracellular PHB content.

### Batch photofermentation assays using ingested pâté olive cake (IPOC)

IPOC is a dark fermented effluent, obtained from the first phase of a two-phase anaerobic digestion process. The effluent was provided by “Frantoio oleario Cassese Domenico”, located in Villa Castelli, Brindisi, Italy. Dark fermented effluent was centrifugated for 30 min at 3800 × g (22 °C) to separate the solid fraction. The suspension was diluted with distilled water in 1:2 *v*/*v* ratio. The concentration of lactic and acetic acid in the IPOC were determined using a Varian Pro Star HPLC chromatograph equipped with an Aminex 87H column maintained at 65 °C, a 20 mL loop for samples injection, a UV detector (l 1⁄4 210) and a refractive index (RI) detector. The eluent was 0.01 N H_2_SO_4_ (Carlo Erba, Milan, Italy) at a flow rate of 0.6 mL min^−1^. The concentrations of metals and other inorganic compounds were determined through inductively coupled plasma–optical emission spectrometry ICP-OES analyzer (Thermo Scientific^™^ iCAP^™^ 7400, US), using IRSA 3010 (conventional acid mineralization) and IRSA A-3020 (determination of chemical elements by spectroscopy emission with plasma source) methods. The pH of IPOC was measured with a pH-meter (XS instruments, Italy), checked before autoclaving, and adjusted to a value of 6.14 after autoclaving. The ammonium concentration was determined with Nessler method (Adessi et al. 2012), using ammonia high range reagent kit HI93733-03 and the HI93733 photometer for ammonium determination (Hanna Instruments, Inc). The PNSB strains were grown using acetic RPN at 24 °C under continuous light until reaching a cell concentration of 400 mg L^−1^. The biomass was centrifugated for 20 min at 2000 × *g* and the pellets re-suspended with the IPOC substrate to a final volume of 100 mL, with an initial cell concentration of 100 mg L⁻^1^ as CDW. The photofermentation batch assay was carried out under anaerobic conditions using rubber-stropped glass bottles (100 mL) equipped with needle to allow the outflow of hydrogen, possibly produced during fermentation. To avoid contamination, each needle was equipped with a 0.2 μm Nucleopore filter (Whatman International, Ltd., Maidstone, England). The 100 mL bottles were kept at 25 °C under a light intensity of 150 μmol (photons) m^−2^ s^−1^. The duration of the experiments was 30 days. Bacterial growth was investigated by determining the BChl *a* at the starting point (T_0_; day 0) and at the end of the experiment (T_30_; day 30). The PHB produced by PNSB was determined at the end (T_30_) of the experiment.

### Analytical methods

Biomass growth was measured in terms of optical density at a wavelength of 660 nm (OD_660_), bacteriochlorophyll *a* (BChl *a)* content, and CDW. Culture turbidity (optical density) was measured spectrophotometrically at 660 nm (OD_660_) using a UV-3100PC UV–vis spectrophotometer (Varian, Mulgrave, Australia). BChl *a* was determined according to Carlozzi and Sacchi (2001). Each sample was centrifugated at 4000 × *g* for 10 min, and the supernatant was removed. BChl *a* was extracted from the cell pellet with acetone/methanol (7:2) ratio. The samples were incubated at 4 °C for 30 min and then centrifugated at 4000 × *g* for 10 min. BChl *a* content was determined by measuring the absorbance at 775 nm (ɛ = 75 mM^−1^ cm^−1^) with a Varian Cary50 UV–visible spectrophotometer (Varian, Mulgrave, Australia). CDW was expressed as the change in dry weights (Δ dry weight) from T_0_ to T_14_, where T_0_ represents the cell dry weight after inoculation, and T_14_ indicates the cell dry weight at the end of the experiments. Determination of CDW was carried out in triplicate using 5 mL culture samples filtered using pre-weighted 0.45 µm membranes (Fisher Scientific International, Inc., Pittsburgh, PA, USA), dried at 105 °C for 3 h, and weighed on an analytical balance (Sartorius AG, Göttingen, Germany).

The intracellular content of poly-β-hydroxybutyrate (PHB) was determined in the form of crotonic acid by HPLC, according to De Philippis et al. (1992b). Briefly, acid cellular hydrolysis was carried out to convert PHB into crotonic acid. At the end of batch assay, a 60 mL sample from each 100 mL bottle was harvested. The sample was centrifugated at 2000 × *g* for 10 min; the pellet was resuspended with 1 mL of distilled water and finally dried at 50 °C overnight. Then, dried cells were weighted, and 3 mL of pure sulfuric acid was added in each screw-cap glass test tubes. Subsequently the tubes were heated at 105 °C for 30 min, cooled on ice for 10 min, and diluted to 3% with distilled water. The solution in each tube was filtered and centrifugated at 16,000 × *g* for 5 min. The supernatant was then analyzed using HPLC for PHB quantification. The crotonic acid concentration was determined using a Varian Pro Star HPLC system, equipped with an Aminex 87H column maintained at 65 °C. The system utilized a 20 µL injection loop, UV detector (λ = 210 nm), and refractive index (RI) detector. The eluent was 0.01 N H₂SO₄ (Carlo Erba, Milan, Italy) at a flow rate of 0.6 mL min⁻^1^. The equations for calculating the PHB yield, determined based on crotonic acid concentration, are provided in detail in Supplementary Material, Appendix 1.

### Transmission electron microscopy (TEM)

TEM observations were performed at the end of the 30-day photofermentation batch experiments on IPOC substrate. Bacterial cells were treated with 2.5% (*w*/*v*) glutaraldehyde at 4 °C, then suspended twice in phosphatase buffer (PBS) 0.1 M, pH 7.0 for 15 min each, and treated with 1% osmium tetroxide, pH 7 for 1 h. Subsequently the samples were suspended with PBS and dried. The samples were then rehydrated in a graded ethanol series (25%, 50%, 70%, 80%, 90%, and 100%) for 10 min each. Spurr’s epoxy resin was used to infiltrate and embed the samples. The Spurr’s epoxy resin was then placed in propylene oxide for 10 min in a solution 2:1 propylene oxide/resin, followed by a 1:1 solution of propylene oxide/resin for minimum of 1 h, and a 1:2 propylene oxide/resin from 1 h to overnight. The samples were cut and post-stained with uranyl acetate and lead citrate. TEM images were acquired with a Philips CM12 transmission electron microscope (Philips Electronics, Amsterdam, The Netherlands) equipped with CRYO-GATAN UHRST 3500 technology and a digital camera.

### Statistical analysis

All the analyses were conducted in experimental triplicates (*N* = 3); a minimum number of three instrumental replicates was always used for each measurement (*n* = 3). To determine whether the results were significantly different, data were analyzed using one-way and two-way analysis of variance (ANOVA) at 95% of the significance, followed by Tukey's honest significance difference (HSD) post-hoc test and Bonferroni post-hoc test. Results were considered statistically significant at *p* < 0.05. Prior to ANOVA, all data were checked for normality assumptions using the D’Agostino-Pearson normality test and for homogeneity of variance using Bartlett’s test. Statistical analysis was performed using GraphPad Prism version 9.00 (California, USA).

## Results

### Strains identification

**Fig. 1:**
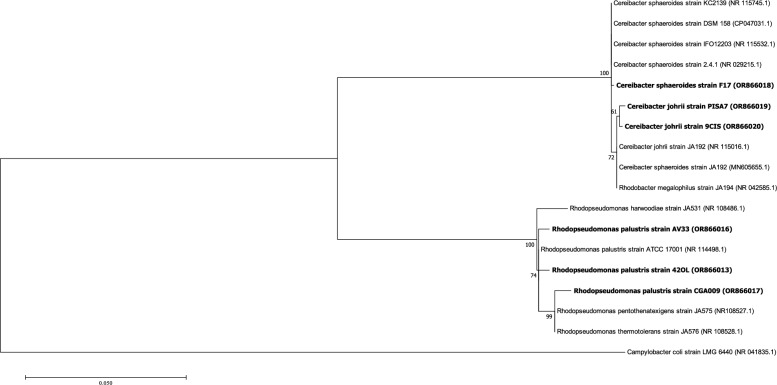
Phylogenetic tree analysis of sequences of the 16S rRNA regions. The numbers given on branches are frequencies (> 50%) with which a given branch appeared in 1000 bootstrap replications. The scale indicates the number of expected substitutions accumulated per site. The tree is rooted with *Campylobacter coli* strain LMG 6440. Type strains used in this study: (*Cereibacter sphaeroides* strain DSM 158^ T^, *Rhodopseudomonas palustris* strain ATCC 17001^ T^, *Cereibacter johrii* JA192^T^). GenBank sequence codes are reported after strain numbers

The phylogenetic tree (Fig. 1) revealed three major clusters: (i) strain F17 which showed a high similarity to the type-strain *Cereibacter sphaeroides* strain DSM 158^ T^; (ii) strain 9Cis and strain PISA 7 showed a high similarity with the type-strain *Cereibacter johrii* strain JA192^T^*;* (iii) and strain 42OL, strain AV33 and strain CGA009 exhibited a high similarity to the type-stain *Rhodopseudomonas palustris* strain ATCC 17001^ T^.

### Batch photofermentation assays using synthetic media

**Fig. 2:**
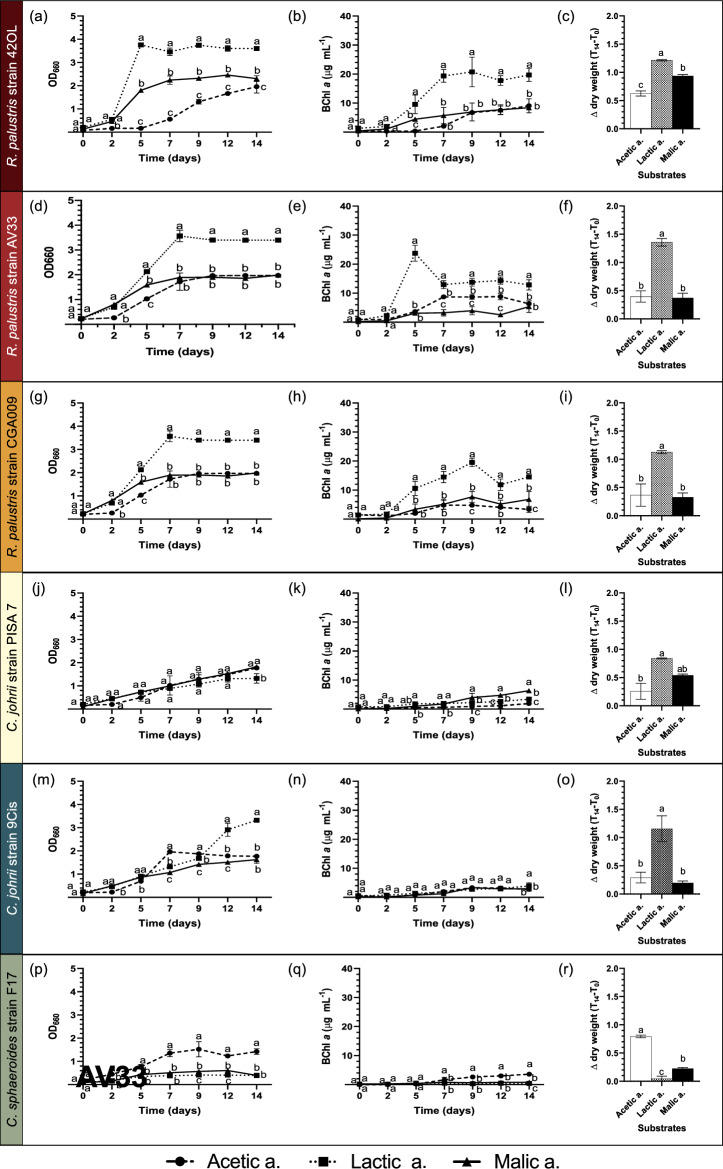
Optical density at 660 nm (OD_660_), bacteriochlorophyll *a* content (BChl* a*) and Δdry weight T_0_-T_14_, representing biomass change from T_0_ to T_14_ days, measured during the growth of *R. palustris* strain 42OL (**a**, **b** and **c**), *R. palustris* strain AV33 (**d**, **e** and **f**) *R. palustris* strain CGA009 (**g**, **h** and **i**), *C. johrii* strain PISA 7 (**j**, **k** and **l**), *C. johrii* strain 9Cis (**m**, **n** and **o**), and *C. sphaeroides* strain F17 (**p**, **q** and **r**) in RPN medium with different carbon sources (acetic ●, lactic _◾_ and malic ▲ acids). Results are the means of three replicates (*N* = 3), with error bars indicating the standard deviation (SD). Differences in the growth of each strain using different carbon sources (acetic, lactic, and malic acids) at each sampling day (OD_660_, BChl* a*) or from T_0_ to T_14_ days (Δdry weight T_0_-T_14_) were evaluated using one-way ANOVA followed by the Turkey’s honest significance test (HSD). Different lowercase letters indicate significant differences (*p* < 0.05)

The behaviors of the six selected strains during photoautotrophic growth on RPN using different single carbon sources (acetic, lactic and malic acids) are represented in Fig. 2. *R. palustris* strain 42OL showed the highest values of OD_660_, BChl *a* and CDW, expressed as Δ dry weight (T_14_-T_0_), in the RPN medium with lactate, followed by the RPN medium enriched with malic acid (Fig. 2a, b, c). The lowest biomass accumulation was obtained with acetate RPN (Fig. 2a; c). *R. palustris* strain AV33 showed the highest value of BChl *a* after 5 days under lactic RPN (Fig. 2d), while no significant differences were observed in the growth of this strain on the RPN with acetic and malic acid in terms of OD_660_, BChl* a* and CDW by the end of the experiment (Fig. 2d, e and f).

*R. palustris* strain CGA009 followed a similar trend to *R. palustris* strain 42OL, showing no significant differences between the RPN with acetic acid and RPN with malic acid, except for the BChl *a* content at the end of the experiment (Fig. 2g, h and i). The highest OD_660,_ BChl *a*, and CDW values were observed in the presence of lactate. *C. johrii* strain PISA 7 showed the most effective growth and metabolic activity when cultured in RPN with lactic acid, as indicated by higher OD_660_, BChl* a* content and CDW after 14 days (Fig. 2j, k and l)*. C. johrii* strain 9Cis showed higher OD_660_ values after 12 days in the RPN medium with lactic acid (Fig. 2m), a trend further supported by the determination of the BChl *a* content and CDW (Fig. 2n and o). *C. sphaeroides* strain F17 exhibited a greater growth on the substrate enriched with acetic acid compared to the other organic acids tested, as evidenced by OD_660_, BChl *a* and CDW measurements (Fig. 2p, q and r).

**Fig. 3:**
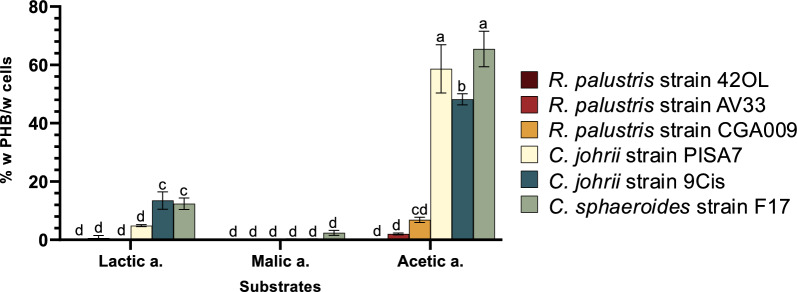
Intracellular poly-β-hydroxybutyrate (PHB) concentration measured at the end of the photofermentation batch assay (14-day) using RPN medium with different carbon sources (acetic, lactic, and malic acids) conducted on *R. palustris* strain 42OL, *R. palustris* strain CGA009, *R. palustris* strain AV33, *C. johrii* strain 9Cis, *C. johrii* strain PISA 7, *C. sphaeroides* strain F17. Results are the means of three replicates (*N* = 3), with error bars indicating the standard deviation (SD). Differences in PHB content among the six different strains using the three different carbon sources on the RPN medium were evaluated using one-way ANOVA followed by the Turkey’s honest significance test (HSD). Different lowercase letters indicate significant differences (*p* < 0.05)

The highest PHB content (Fig. 3), volumetric production and daily productivity (Table 3) were detected in *C. sphaeroides* strain F17 (65.45 ± 6.08% w PHB/w cells), followed by *C. johrii* strain PISA 7 (58.64 ± 8.28% w PHB/w cells) and *C. johrii* strain 9Cis (48.18 ± 1.92% w PHB/w cells). These strains also demonstrated notable PHB accumulation when cultured in the synthetic medium with lactic acid. On the other hand, the three *R. palustris* strains accumulated low amounts of intracellular PHB, and only when grown with acetic acid as carbon source. In particular, *R. palustris* strain CGA009 showed a PHB content of 6.87 ± 0.90% w PHB/w cells when grown on RPN with acetic acid, while *R. palustris* AV33 showed a content of 2.05 ± 0.33% w PHB/w cells in the same growth condition (Fig. 3). Similarly, the PHB volumetric production and daily productivity (Table 3) were very low for all the *R. palustris* strains.

### Growth capability and PHB production using IPOC

**Fig. 4:**
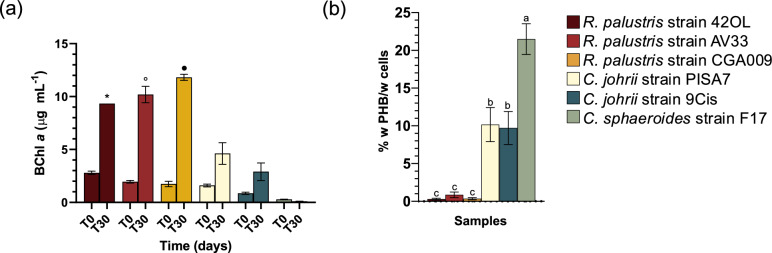
**a** Bacteriochlorophyll *a* content (BChl* a*) measured after inoculation (T_0_; day 1) and at the end of the experiment (T_30_; day 30) using *R. palustris* strain 42OL, *R. palustris* strain AV33, *R. palustris* strain CGA009, *C. johrii* strain PISA 7, *C. johrii* strain 9-Cis, and *C. sphaeroides* strain F17 grown on IPOC substrate. Results are the means of three replicates (*N* = 3), with error bars indicating the standard deviation (SD). Differences in BChl *a* content between time points (T_0_ and T_30_) were evaluated using two-way ANOVA followed by Bonferroni’s post-hoc test. Different symbols indicate significant differences (*p* < 0.05) in BChl *a* content between T_0_ and T_30_ for each strain. **b** Intracellular poly-β-hydroxybutyrate (PHB) contents measured at the end of the 30-day photofermentation batch experiment on IPOC substrate using *R. palustris* strain 42OL, *R. palustris* strain AV33, *R. palustris* strain CGA009, *C. johrii* strain 9Cis, *C. johrii* strain PISA 7, *C. sphaeroides* strain F17. Results are the means of three replicates (*N* = 3), with error bars indicating the standard deviation (SD). Differences in PHB content among the six different strains were evaluated using one-way ANOVA followed by the Turkey’s honest significance test (HSD). Different lowercase letters indicate significant differences (*p* < 0.05)

The composition of IPOC was shown in Table 2. The dark fermented effluent contained 0.73 g/L of acetic acid, 1.37 g/L of NH_4_^+^, a C/N ratio of 0.25 and a pH of 6.14. The compositions of metals and ions in IPOC showed a high content of K (221.54 ± 15.4 g L^−1^), followed by Na (8.50 ± 0.18 g L^−1^), Ca (2.49 ± 0.86 g L^−1^), Fe (1.03 ± 0.01 g L^−1^), S (0.25 ± 0.07 g L^−1^), Mg (0.22 ± 0.03 g L^−1^) and Si (0.19 ± 0.01 g L^−1^) (Table 2). The suitability of IPOC for supporting the growth and PHB production of the six selected strains was investigated. *R. palustris* strain 42OL, *R. palustris* strain AV33 and *R. palustris* strain CGA009 showed a significant increase of BChl *a* content from the beginning (T_0_; day 1) to the end (T_30_; day 30) of the batch fermentation experiments (Fig. 4a). In contrast, *C. sphaeroides* strain F17, *C. johrii* strain PISA 7 and *C. johrii* strain 9Cis did not exhibit any variation of their BChl *a* content. On the other hand, the PHB content at the end of the experiment was significantly higher in the batch fermentation experiment inoculated with *C. sphaeroides* strain F17 (21.48 ± 2.43% w PHB/w cells), followed by *C. johrii* strain PISA 7 (10.16 ± 2.26% w PHB/w cells) and *C. johrii* strain 9Cis (9.71 ± 2.19% w PHB/w cells) (Fig. 4b). Conversely, strains belonging to the *R. palustris* species showed a PHB content lower than 1.02% w PHB/w cells. The PHB volumetric production and daily productivity (Table 3) were in accordance with the trend described.

**Table 2:** Composition of ingested pâté olive cake (IPOC)

Compounds	Concentration (g L^−1^)
Lactic acid (g L^−1^)	Nd
Acetic acid (g L^−1^)	0.73 (± 0.01)
NH_4_^+^	1.37 (± 0.31)
B	Nd
Ca	2.49 (± 0.86)
Cd	Nd
Co	Nd
Cr	Nd
Cu	Nd
Fe	1.03 (± 0.01)
K	221.54 (± 15.4)
Mg	0.22 (± 0.03)
Mn	Nd
Mo	Nd
Na	8.50 (± 0.18)
Ni	Nd
S	0.25 (± 0.07)
Si	0.19 (± 0.01)
Zn	Nd

Results are expressed as mean ± standard deviation (SD), *n* = 3

^*^nd not detected

**Table 3:** Volumetric PHB final production (mg_PHB_ L_cult_^−1^) and daily productivity (mg_PHB_ L_cult_^−1^ d^−1^) for all strains grown on RPN synthetic media containing acetate, lactate or malate as carbon sources and on ingested pâté olive cake (IPOC)

Substrate →	Acetate		Lactate		Malate		IPOC	
Strain ↓	mg_PHB_ L_cult_^−1^	mg_PHB_ L_cult_^−1^ d^−1^	mg_PHB_ L_cult_^−1^	mg_PHB_ L_cult_^−1^ d^−1^	mg_PHB_ L_cult_^−1^	mg_PHB_ L_cult_^−1^ d^−1^	mg_PHB_ L_cult_^−1^	mg_PHB_ L_cult_^−1^ d^−1^
*R. palustris* strain 42OL	1.28 (± 0.34)	0.09 (± 0.02)	0.27 (± 0.05)	0.02 (± 0.01)	0.37 (± 0.11)	0.03 (± 0.01)	1.60 (± 1.18)	0.11 (± 0.08)
*R. palustris* strain AV33	17.27 (± 2.91)	1.23 (± 0.21)	1.18 (± 0.90)	0.08 (± 0.06)	0.38 (± 0.34)	0.03 (± 0.02)	6.41 (± 2.74)	0.54 (± 0.20)
*R. palustris* strain CGA009	40.69 (± 6.13)	2.91 (± 0.44)	0.72 (± 0.08)	0.05 (± 0.00)	0.21 (± 0.14)	0.02 (± 0.01)	2.39 (± 0.85)	0.17 (± 0.06)
*C. johrii* strain 9Cis	339.73 (± 7.87)	24.26 (± 0.56)	70.47 (± 39.00)	5.03 (± 2.78)	1.75 (± 1.06)	0.13 (± 0.08)	68.70 (± 18.42)	4.83 (± 1.85)
*C. johrii* strain PISA 7	373.89 (± 55.11)	26.71 (± 3.94)	25.89 (± 4.19)	1.85 (± 0.30)	1.71 (± 1.31)	0.12 (± 0.09)	73.44 (± 11.78)	4.97 (± 0.98)
*C. sphaeroides* strain F17	399.50 (± 54.77)	28.54 (± 3.91)	14.48 (± 1.82)	1.03 (± 0.13)	5.10 (± 2.38)	0.36 (± 0.17)	151.98 (± 21.05)	10.85 (± 1.50)

Results are expressed as mean ± standard deviation (SD), *n* = 3

**Fig. 5:**
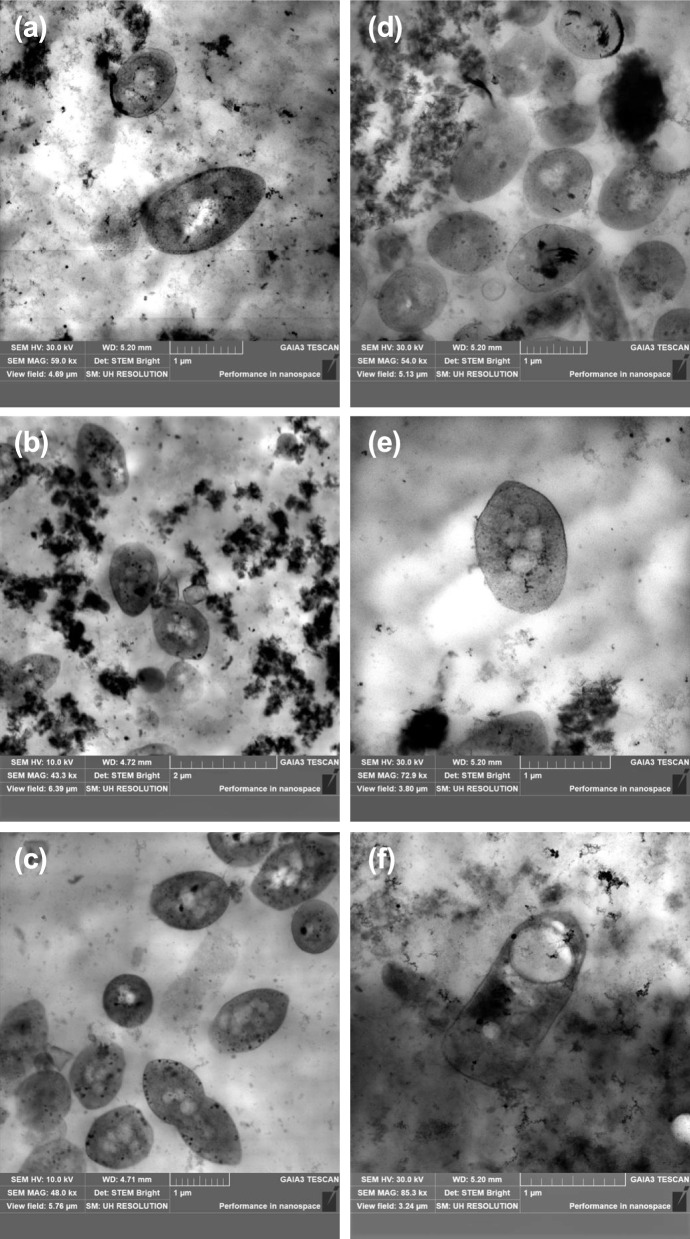
Transmission electron microscopy images of the inclusion bodies obtained from batch photofermentation experiments conducted using *R. palustris* strain 42OL (**a**), *R. palustris* strain AV33 (**b**), *R. palustris* strain CGA009 (**c**), *C. johrii* strain PISA 7 (**d**), *C. johrii* strain 9Cis (**e**) and *C. sphaeroides* strain F17 (**f**) at the end of the 30-day photofermentation batch experiments on IPOC substrate

TEM observations, conducted after 30 days, revealed the presence of small granules of PHB in the cytoplasm as insoluble inclusions across all analyzed samples (Fig. 5). Among the *R. palustris* species, strain 42OL showed cells 0.86 ± 0.20 µm wide with granules with 0.14 ± 0.05 μm of diameters (Fig. 5a). Instead, *R. palustris* strain AV33 displayed a smaller cell size of 0.76 ± 0.28 µm wide with inclusion bodies size of 0.17 ± 0.04 μm (Fig. 5b). *R. palustris* strain CGA009 exhibited large cell size (1.11 ± 0.33 µm wide) and similar PHB granules size of 0.16 ± 0.04 μm (Fig. 5c). *C. johrii* strain PISA 7 exhibited a cell size of 1.23 ± 0.35 µm wide, with a comparatively larger size of PHB of 0.31 ± 0.02 μm (Fig. 5d). On the other hand, *C. johrii* strain 9Cis displayed a larger cell size of 1.45 ± 0.32 µm wide, with PHB granules measuring 0.24 ± 0.05 μm (Fig. 5e). Meanwhile, C. *sphaeroides* strain F17 showed a cell size of 1.44 ± 0.35 µm wide, and its PHB particles were notably larger 0.59 ± 0.05 μm than the other PNSB inclusions bodies analyzed (Fig. 5f).

## Discussion

The application of PNSB for PHB production has recently been expanded to strains originally investigated for hydrogen production. Their use for PHB production is supported by the knowledge previously gained regarding their physiological properties, metabolic traits, optimal growth conditions, biomass production, and carbon-to-biomass conversion efficiency during hydrogen production experiments. *R. palustris* strains 42OL, AV33 and CGA009 were primary studied for hydrogen production (Bianchi et al. 2010; Adessi et al. 2012, 2018). Despite they are members of the same cluster and all show high adaptability to different substrates, the results obtained in this study clearly demonstrated a more efficient substrate assimilation when lactic acid was the C source, resulting in rapid cell growth. These results were also supported by the work of Dipasquale et al. (2015), where the authors found that lactate was the preferred substrate for high yield hydrogen production in combined dark-photo fermentation systems using *R. palustris* 42OL.

Among the *R. palustris* strains*,* the highest content of PHB was produced by the strain CGA009, which was associated with the lowest BChl *a* content, when acetate was used as carbon source. Indeed, the results obtained with *R. palustris* strain CGA009 showed that this strain converts acetate into PHB rather than increasing biomass, as previously noted by Carlozzi et al. (2019a) and Liebergesell et al. (1991) using *Rhodopseudomonas* sp. S16-VOGS3 and *R. palustris* 1850, respectively. Similar findings were also documented by (Wu et al. 2012), utilizing *R. palustris* WP3-5, where acetate was identified as the most favorable substrate for PHB synthesis.

The low PHB accumulation by the *R. palustris* strains 42OL grown using synthetic media with different carbon sources was also confirmed by other studies, where the bacterium was cultured indoors and outdoors, using freely suspended or immobilized cells (De Philippis et al. 1992a; Vincenzini et al. 1997; Carlozzi and Sacchi 2001). Conversely, *C. sphaeroides,* has been extensively documented for its ability to produce more PHB than *R. palustris* species (Monroy and Buitrón 2020)*.* Examining the substrate preferences, our results suggested that *C. sphaeroides* strains F17 grown in the RPN medium with malate as a carbon source produced lower PHB compared to the results achieved by Yigit et al. (1999), 2.40% and 19.8% w PHB/w cells, respectively. In contrast, the results obtained using lactate were very similar to those reached by Khatipov et al. (1998) using *C. sphaeroides* RV.

In addition, it is also remarkable that the results obtained from growing the same strain with acetic acid in RPN medium revealed that this carbon source not only yielded the highest PHB content but also promoted the highest cell growth, demonstrating a significant advantage over all other substrates. One plausible explanation for this phenomenon is the easy conversion of acetate into acetyl-CoA, a key precursor in the formation of PHB, as proposed by Karthikeyan et al. (2015). In particular, comparing the PHB accumulated by *C. sphaeroides* strain F17 using acetate as carbon source with other studies (Liebergesell et al. 1991; Khatipov et al. 1998; Kemavongse et al. 2008; Kim et al. 2011), it worth mentioning that its production is very promising. Especially, PHB content obtained in this study by employing NH_4_Cl, yeast extract, and acetate (65.45% w PHB/w cells), are higher than those reported by Khatipov et al. (1998) and Kim et al. (2011); which were 40% and 54% w PHB/w cells, respectively, obtained using ammonium and ammonium sulfate. However, a comparable yield was achieved by Liebergesell et al. (1991), with values of 63% and 69.9% w PHB/w cells in *C. sphaeroides* Y and *C. sphaeroides* 17,023, respectively, while Kemavongse et al. (2008) obtained a PHB yield of 87% w PHB/w cells using *C. sphaeroides* U7. Therefore, according to Khatipov et al. (1998) acetic acid constitutes the main carbon source promoting PHB accumulation, significantly enhanced under stress conditions, in *C. sphaeroides*, (Touloupakis et al. 2021).

Although several studies have been conducted on *Rhodopseudomonas* sp. and *C. sphaeroides*, limited information is available on the specie *C. johrii* (Hördt et al. 2020). The *C. johrii* strains PISA 7 and 9Cis exhibit high similarity across most of the sequenced regions of the 16S rRNA gene; however, their PHB production differs significantly depending on the different carbon sources. In particular, *C. johrii* strain PISA 7 displayed a significantly higher accumulation of PHB when growth on RPN supplemented with acetic acid, whereas PHB accumulation was comparatively lower when lactic acid was used as the carbon source, in contrast to the *C. johrii* strain 9Cis. These differences were also confirmed by growth profiles results, expressed in terms of OD_660_, BChl *a* and CDW. *C. johrii* strain PISA 7 exhibited higher BChl *a* and CDW contents when grown on lactic and malic acid as carbon sources. In contrast, *C. johrii* strain 9Cis showed higher growth performances on the lactic acid-enriched substrate.

Since malate and lactate can easily enter the TCA cycle and satisfy the cell energy needs, unlike acetate, it would be interesting to investigate the potential applications of these two new strains of *C. johrii* not only for the PHB accumulation but also for hydrogen production processes (Kars and Gündüz 2010).

Overall, metabolic profiles indicate different nutrient aptitudes among the selected six strains using culture media enriched with malic, acetic and lactic acid. The *R. palustris* strains showed similar metabolic pattern, with lactic acid identified as the optimal carbon source for biomass production. However, despite stable growth across different single carbon sources, *R. palustris* strains displayed low PHB accumulation, likely due to unsuitable growth conditions for promoting PHB production. Therefore, a potential strategy to enhance PHB production is to use a multi-steps cultivation process with different nutrient conditions, starting with nutrient-sufficient conditions, followed by a stage of nitrogen or magnesium limitation (Corneli et al. 2016), concluding with a final stage of sulfur deficiency (Carlozzi et al. 2019a). Conversely, under the same culture conditions, *C. johrii* strain PISA 7 and 9Cis, as well as *C. sphaeroides* strain F17, exhibited limited growth but enhanced PHB accumulation. This preference for PHB production over biomass synthesis may reflect species-specific bacterial adaptations. This shift may result from a complex interplay of factors, including genetic diversity, enzyme specificity, regulatory networks, metabolic pathway flexibility, and evolutionary processes (Amadu et al. 2021). All these factors contribute to the ecological niche specialization of bacterial strains and their capacity to adapt to diverse environments.

Despite the strains exhibited a cluster-associated trend in terms of nutritional traits, growth aptitudes, and PHB productions, their characterization in pure culture and synthetic media provides several advantages, such as identifying favorable conditions to promote PHB production. On the contrary, the bioconversion of complex agro-industrial waste into valuable products requires multiple investigations into both strain capabilities and substrate composition to define the optimal conditions for successful bioconversion. In this study, based on the insights obtained from the synthetic batch experiment, the same strains were fed with ingested pâté olive cake (IPOC) to produce PHB through the photo-fermentative process.

The dark fermented effluent from the first phase of a two-phase anaerobic digestion process contained 0.73 g/L (12.00 mM) of acetic acid. Although this concentration is significantly lower than the recommended 50 mM needed to promote efficient PHB production (Monroy and Buitrόn, 2020), the results obtained with the strains *C. johrii* stain PISA 7, 9Cis, and *C. sphaeroides* F17 are very promising. In contrast, strains of the *R. palustris* genus displayed a significant increase in BChl *a* content, favoring biomass production rather than PHB accumulation. The volatile fatty acids (VFAs) content in the dark fermented effluent is similar to that reported by Corneli et al. (2016) using ensiled olive pomace, as well as the PHB produced by the strain *C. johrii* stains PISA 7 and 9Cis. The lower VFA concentration in both these studies, compared to that of the substrate used in Padovani et al. (2016), is likely due to the high contents of phenols and furans in the solid fraction of IPOC and ensiled olive pomace (eOP), which are commonly identified as inhibitors of the acidogenesis phase of anaerobic digestion (Vavouraki et al. 2020; Caroca et al. 2021). Another important operating parameter that could affect PHB production is the C/N ratio. Indeed, the high content of NH_4_^+^ (1.37 g/L) in IPOC substrate reduced this ratio to 0.25, a value that is 120-fold lower than the recommended level for effective PHB synthesis (Monroy and Buitrόn, 2020).

It is well known that the limitation or the absence of elements such as phosphorus and magnesium enhance PHB production (Corneli et al. 2016; Montiel-Corona and Buitrón, 2021; Sali and Mackey 2021). Results of ICP-OES analysis (Table 2) indicated that the Mg^2+^ content in the acetic RPN medium is about 79.8% higher than those in the IPOC substrate, acting as a limiting factor that could trigger PHB accumulation. However, this Mg^2+^ concentration is higher than that reported in ensiled olive pomace by Corneli et al. (2016). In literature, there is a lack of data regarding the influence of potassium, sodium and calcium on PHB production by PNSB. In IPOC, potassium concentrations were 270 times greater than those in the acetic RPN medium, sodium 53 times higher, and calcium levels were over 33 times higher than in the synthetic medium. Such elevated concentrations of calcium, sodium and potassium may affect cellular osmotic balance and could potentially reduce the ability of PNSB to produce PHB (Obruca et al. 2017). Furthermore, the high concentration of trace metals, such as Fe, which is more than 200 times higher in IPOC than in acetic RPN medium, can also negatively affect the PHB yield (Monroy and Buitrón, 2020).

Overall, different strains adopted different metabolic strategies when fed with the IPOC substrate, likely driven by highly specialized metabolic pathways shaped by the selective pressures of their ecological niches. Strains belonging to *R. palustris* (strains 42OL, CGA009 and AV33) used the IPOC substrate through a replicative metabolism, increasing their biomass during the incubation. In contrast, the *C. johrii* strain PISA 7 and 9Cis, along with *C. sphaeroides* F17, promoted PHB metabolism by converting carbon sources into precursors that were then polymerized to form PHB granules within the cells. The district metabolic strategies adopted by the different strains in this study were confirmed by transmission electron microscopy (TEM) micrographs. *R. palustris* strains exhibited smaller cells with small inclusion bodies, while *C. johrii* strains PISA 7 and 9Cis displayed larger cells containing PHB granules twice the size of those observed in *R. palustris.* Notably, *C. sphaeroides* strain F17 showed the largest cell size and the most substantial PHB granules among all the examined PNSB, providing comprehensive insight into their distinctive metabolic behaviors. Furthermore, the volumetric production, daily productivity and PHB content achieved in this study with *C. sphaeroides* strain F17 (21.48 ± 2.43% w PHB/w cells) represent the basis for a promising biotechnological application, exceeding previously reported content for untreated IPOC, including Corneli et al. (2016; 11.54 ± 0.64% w PHB/w cells), Padovani et al. (2016; 9.0% w PHB/w cells), and Carlozzi et al. (2019b; 15.7% w PHB/w cells). These findings highlight the remarkable capability of *C. sphaeroides* strain F17 of bioconverting complex waste substrates. The differences in PHB accumulation observed for *C. sphaeroides* strain F17 comparing the synthetic media and IPOC may be linked to the specific metabolic behaviors of this strain or to the effects of certain IPOC-derived compounds that influence PHB synthesis through distinct mechanisms. These findings highlight the need for further investigation into the molecular and biochemical mechanisms underlying strain-specific responses to complex substrates such as IPOC.

The current work used an integrate approach of phylogenetic analysis, metabolic characterization and morphological evaluation to identify strains suitable for waste valorization through PHB accumulation. While photosynthetic bacteria offer standard advantages, such as reduced aeration requirements and the ability to utilize a wide range of waste substrates, phylogenetic analysis and metabolic characterizations conducted on synthetic media and in pure culture provide predictive insights into their performance on complex substrates, such as IPOC. Furthermore, the morphological characterization of the strains using TEM observation conducted directly on the selected PNSB strains grown on the complex IPOC substrate, offers a significant advantage. This approach provides valuable information on cell dimensions and inclusion bodies sizes, which are strictly associated to the PHB yields. It also highlights the adaptability of the strains during growth in IPOC, which is among the most inhibitory substrates for microbial growth in the Mediterranean basin due to its high content of phenolic and antimicrobial compounds. These findings enhance our understanding of strain-specific responses and their capacity for PHB accumulation.

In addition, growing these strains on the IPOC substrate also presents several challenges. High concentrations of ammonium and phenolic compounds in IPOC can inhibit acetic acid production, thereby limiting PHB accumulation. Additionally, elements such as iron, potassium, and calcium present in IPOC may interfere with PHB synthesis, highlighting the necessity for targeted optimization strategies to enhance metabolic performance. Despite these limitations, the PHB yield achieved in this study with *C. sphaeroides* strain F17 highlights its suitability as a valuable candidate for producing PHB and other substances of economic interests.

## Conclusion

The bioconversion of olive mill wastes into high-value bioproducts, specifically PHB, using six strains from three distinct species (*R. palustris*, *C. sphaeroides*, and *C. johrii*), has provided significant insight into their metabolic capabilities and bioconversion potential. The dual aptitudes of these strains, characterized by their replication strategies and PHB production capabilities, along with their distinct morphological traits, has provided paramount information for identifying promising candidates for further investigation. Among these, *C. sphaeroides* strain F17 and the novel *C. johrii* strains 9Cis and PISA 7 have emerged as interesting candidates for future research.

Future laboratory-scale studies should focus on optimizing the growth conditions and PHB production of these strains by investigating the effects of key parameters, such as temperature, pH, and nutrient concentrations. Parallel efforts should focus on the optimization of treatments for the IPOC substrate to mitigate potential inhibitory factors, including high concentrations of phenols, iron, calcium, potassium, and nitrogen, while enhancing the availability of acetic acid.

These laboratory-scale efforts must finally be validated on the pilot scale, where productivity and production costs are assessed to establish the feasibility of future industrial applications. This comprehensive approach will lay the foundations for the development of an efficient and economically viable biorefinery process.

## Supplementary Information

Supplementary material 1.

## Data Availability

Data are available upon request to the authors.
