# Supplementary material for: Photofermentative production of poly-β-hydroxybutyrate (PHB) by purple non-sulfur bacteria using olive oil by-products
Source: Bioresour Bioprocess. 2025 Mar 24;12(1):25. doi: 10.1186/s40643-025-00856-x (PMC11933499; doi:10.1186/s40643-025-00856-x)
Supplement: Supplementary file 1 — Supplementary material 1. [file 40643_2025_856_MOESM1_ESM.pdf]

Photofermentative production of poly-β-hydroxybutyrate (PHB) by purple non-sulfur bacteria using olive oil by-products

Gianmarco Mugnai[1,2], Luca Bernabò[1], Giulia Daly[1], Elisa Corneli[1,3], Alessandra Adessi[1]*

[1] Department of Agriculture, Food, Environment and Forestry (DAGRI), University of Florence, Piazzale delle Cascine, 18, Florence, 50144, Italy.

[2] Department of Agricultural, Food and Environmental Sciences, University of Perugia, Borgo XX Giugno, 74, Perugia, 06121, Italy.

[3] PhotoB. Srl, Via Montecalvi, 3, 50026, San Casciano in Val di Pesa, Florence, Italy.

*corresponding author: Alessandra Adessi, Department of Agriculture, Food, Environment and Forestry (DAGRI), University of Florence, Via Maragliano 77, Florence, 50144, Italy; alessandra.adessi@unifi.it

Appendix 1: calculation of polyhydroxybutyrate (phb) content based on crotonic acid concentration.

This calculation aims to estimate the percentage of PHB in a sample, based on the concentration of crotonic acid obtained from High-Performance Liquid Chromatography (HPLC) analysis. This approach involves a stoichiometric conversion and adjustment for water loss.

Methodology

1. **Assumptions and Definitions:**

   -Let X be the concentration of crotonic acid detected in the sample (mg/L)
   -Molar mass value:
   Crotonic acid ($MW_{crotonic\ acid}$) = 86.09 g/mol.
   3-hydroxybutyric acid ($MW_{3\text{-}hydroxybutyric\ acid}$) = 104.11 g/mol.

2. **Calculation step:**
   - Calculate moles of crotonic acid based on X

$$\text{Moles of crtonic acid} = \frac{X}{\text{MW crotonic acid}}$$

   - Assume moles of 3-hydroxybutyric acid are equivalent:

$$\text{Moles of 3hydroxybutyric acid} = \frac{X}{\text{MW crotonic acid}}$$

- Calculate the concentration of 3-hydroxybutyric acid in mg/L:

$$\text{Concentration of } 3-\text{hydroxybutyric acid} = \frac{\text{X} \times \text{MW } 3-\text{hydroxybutyric acid}}{\text{MW crotonic acid}}$$

- Adjust for Sample Volume
  Assuming the sample volume was 100 mL (as opposed to 1L), adjust the concentration accordingly:

$$\text{Mass of 3hydroxybutyric acid in 100 mL} = \frac{\text{X} \times \text{MW } 3-\text{hydroxybutyric acid}}{\text{MW crotonic acid}} + \frac{100\text{ ml}}{1000\text{ ml}}$$

- Adjust for water loss
  using a proportion to account for water molecule (half a water molecules):

  0.043 : 0.036 = Mass of 3-hydroxybutyric acid in 100 mL : Y

Solving for Y (mg of PHB in 100 ml), the corrected PHB mass:

$$Y = \frac{0.036 \times \text{Mass of } 3-\text{hydroxybutyric acid in 100 ml}}{0.043}$$

- Calculate PHB content on Dry Cell Weight basis:
  Given a dry cell weight of cells W (e.g., 165 mg), calculate the PHB %:

$$\text{PHB \% (dry weight)} = \frac{\text{Y}}{\text{W}} \times 100$$

This calculation is a stoichiometric approach for estimating PHB content based on crotonic acid concentration and is suitable for conditions where crotonic acid is a reliable marker for PHB breakdown.
